# Closing the False Divide: Sustainable Approaches to Integrating Mental Health Services into Primary Care

**DOI:** 10.1007/s11606-016-3967-9

**Published:** 2017-02-27

**Authors:** Kurt Kroenke, Jurgen Unutzer

**Affiliations:** 1 VA HSR&D Center for Health information and Communication, Indianapolis, IN USA; 2 Department of Medicine, Indiana University School of Medicine, Indianapolis, IN 46202 USA; 3 Regenstrief Institute, Inc, 1101 West 10th St, Indianapolis, IN 46202 USA; 4 Department of Psychiatry and Behavioral Sciences, University of Washington, Seattle, WA USA

## Abstract

Mental disorders account for 25% of all health-related disability worldwide. More patients receive treatment for mental disorders in the primary care sector than in the mental health specialty setting. However, brief visits, inadequate reimbursement, deficits in primary care provider (PCP) training, and competing demands often limit the capacity of the PCP to produce optimal outcomes in patients with common mental disorders. More than 80 randomized trials have shown the benefits of collaborative care (CC) models for improving outcomes of patients with depression and anxiety. Six key components of CC include a population-based approach, measurement-based care, treatment to target strategy, care management, supervision by a mental health professional (MHP), and brief psychological therapies. Multiple trials have also shown that CC for depression is equally or more cost-effective than many of the current treatments for medical disorders. Factors that may facilitate the implementation of CC include a more favorable alignment of medical and mental health services in accountable care organizations and patient-centered medical homes; greater use of telecare as well as automated outcome monitoring; identification of patients who might benefit most from CC; and systematic training of both PCPs and MHPs in integrated team-based care.

## PRIMARY CARE INTEGRATION RATIONALE AND BARRIERS

Worldwide, the prevalence of major depression and anxiety disorders is 5.6% and 4.0%, respectively, and mental disorders account for nearly 25% of all health-related disability.^1^ Depression and anxiety are each present in about 10% of primary care patients in the US^2^ and are the first and fifth most common causes of years lived with disability (YLDs) among all diseases.^3^ Indeed, depression and anxiety alone account for as many YLDs in the US as do chronic obstructive pulmonary disease, diabetes, Alzheimer disease, ischemic heart disease, stroke, and chronic kidney disease combined.

Of all mental health (MH) visits, more are to primary care providers (PCPs) than to psychiatrists, and the PCP proportion has been rapidly increasing. Of the 22 visits per 100 persons in the US population to psychiatrists and PCPs that resulted in a mental health diagnosis in 2010, 60% were to a PCP (compared to 54% in 1995); of the 35 visits per 100 persons that resulted in a psychotropic medication prescription, 77% were to a PCP (compared to 67% in 1995).^4^ A majority of patients are treated for depression exclusively in primary care (73%) with fewer patients treated by psychiatrists (24%) or other mental health professionals (13%).^5^ Although PCPs provide the majority of MH visits, these visits only make up a small proportion of their overall work: 3% and 2% of PCP encounters are coded for a primary diagnosis of depression and anxiety compared with 41% and 27% of psychiatrist visits.^4^

Besides prevalence, other factors mandating a principal role for PCPs include a shortage of mental health specialists and the reluctance of some patients to accept a mental health referral. Fewer than one in four adults with a diagnosable mental disorder receive care from a mental health professional in any given year.^6^ Many patients prefer receiving treatment from providers with whom they already have an established health care relationship.^7^ Barriers include: (1) insufficient training and/or interest of some PCPs in managing mental disorders; (2) the brevity of primary care visits; (3) the competing demands of preventive care and treatment of comorbid medical conditions; (4) reimbursement systems that carve out mental health care or constrain payment to PCPs for adequate treatment of mental disorders; (5) confidentiality concerns of MH providers or patients that impede sharing of notes; (6) disagreements among different types of MH specialists on which providers should be integrated and what roles they should play

## KEY COMPONENTS OF EFFECTIVE INTEGRATION

Because most trials of integrated care have tested multi-component treatments, the evidence supports these bundled interventions as a whole rather than their discrete components (Table 1). The key components outlined in Table 2 are distilled from more comprehensive reviews.^7^^–^ 17

**Table 1:** Six Key Components of Integrating Mental Health into Primary Care

Component	Comment
Population based	•Identify and track all patients with a particular disorder • Difficult without registry and/or care manager
Measurement based	• Screening and severity assessment • Regular treatment outcome monitoring
Treatment to target	• Regularly monitor severity of the disorder • Focus on treating to desired level of the outcome rather than a specific type or dose of drug or psychotherapy • Consult mental health specialist and/or adjust or change treatment if not improving
Care management	• Often nurse (or social worker, pharmacist, etc.) • Psychoeducation, patient registry, systematic tracking of adherence and clinical response, coordinating care among patient, primary care provider and mental health specialist
Psychiatric supervision	• Regular (usually weekly) meetings with care manager to review patients who are not improving as expected • Occasional consultative session with selected patients • Facilitated mental health referral for more complex patients
Brief psychological therapies	• Delivered in-clinic or by telephone or tele-video • Administered by trained behavioral health specialist (e.g., nurse, clinical social worker, psychologist, other licensed counselor) • Limited number of sessions focused on simpler treatments (e.g., behavioral activation, problem-solving)

**Table 2:** **Practical Strategies for Smaller Primary Care Practices**

No.	Strategy
1	Use *self-administered measures* not only for screening but also to monitor and adjust therapy
2	Assess *suicidality* efficiently (e.g., P4 screener) to stratify referral urgency and develop practice protocols for the assessment, triage, and follow-up of patients at risk for suicide
3	Track *key treatment metrics* (symptom response, adherence, side effects) in home-based fashion (e.g., secure website, telephonically, HIPPA-compliant texting or e-mails, etc.)
4	Acquire skills in *brief therapy techniques* (e.g., behavioral activation; motivational interviewing; problem-solving treatment) and train other practice members as appropriate (e.g., social workers or nurses) in such skills
5	Direct patients to *self-management resources* (including web-based) to complement therapy
6	Educate patients with milder symptoms or in remission how to *detect worsening and seek care; establish relapse prevention plans for patients in remission*
7	Train *least costly office staff* for simple tasks (screening, education, monitoring depressive symptom response and treatment adherence, referral)
8	Establish a *list/registry* of patients who have initiated treatment for common mental disorders and a protocol for reviewing this list on a regular basis to identify patients who need changes in treatment, consultation, or a higher level of care (e.g., specialty referral)

1. *Population-based* care for a particular disease means systematic efforts by a health care system to identify all of its patients with that disease, provide appropriate treatment, and track outcomes. In the current context of primary care, this is difficult to do without a disease registry and care manager. Electronic health records can facilitate the process but not without additional programming or resources to identify patients and track adherence, follow-up, and clinical outcomes. A challenge for primary care is to determine how many medical and mental health conditions can be effectively tracked from a population-based care approach. Since the number of common conditions is substantial, prioritization of diseases and integration of registries and care management resources is essential. For example, depression is a priority because untreated depression is not only prevalent and disabling but also interferes with the care of other chronic medical conditions.^18^
2. *Measurement-based* care for some medical conditions is based upon laboratory or physiological measurements (diabetes, hyperlipidemia, hypertension) or objective measures coupled with patient symptoms and function (e.g., cardiovascular, pulmonary, and neurological diseases). For mental disorders like depression and anxiety, patient-reported outcomes (PROs) are the predominant measure guiding therapy. The ideal clinical PRO should be brief, self-report, public domain, multi-purpose (i.e., effective for screening, severity assessment, and monitoring treatment response), easy to score and interpret, and available in multiple languages.^19^ The Patient Health Questionnaire family of scales (e.g., PHQ-9, GAD-7) and the Patient-Reported Outcomes Measurement Information System (PROMIS) scales are two examples of PROs that meet most or all of these pragmatic criteria and are accessible at www.phqscreeners.com and www.assessmentcenter.net.
3. *Treatment to target* means regularly monitoring severity of the disorder and intensifying, changing or augmenting treatment based upon target thresholds. As an example, a 3- to 5-point change on the PHQ-9 is clinically significant; a ≥50% decrease and a score <10 typically signify a treatment response and a score <5 a remission. Scores should not be used in isolation but integrated with the patient’s global sense of improvement (“Are you better, the same, or worse?”), occupational and social functioning, and duration of symptoms. A treat-to-target approach is also not wedded to a particular therapy but instead tailors the type and dose of treatment to patient preferences, treatment availability and tolerance, comorbid medical and mental disorders, and clinical response. It is essential, however, that therapies are evidence-based and adjusted if patients are not improving as expected, which requires a practice strategy to incorporate measurement (component 2) in a systematic way to monitor treatment response.^12^
4. *Care management* involves a clinician (most commonly a nurse but in some settings a pharmacist, social worker, medical assistant, or other health care professional) with dedicated time to follow patients with particular disorders within a practice or group of practices. Common elements include maintaining a disease registry, providing disease-based education, tracking treatment adherence and clinical response, and triangulating care among three relevant parties—the patient, the PCP, and a behavioral health specialist such as a clinical psychologist or psychiatric consultant. Many patient contacts can be achieved telephonically, enhancing convenience and cost-effectiveness. In small or rural practices with fewer resources, individuals without specialized mental health training can perform telephone monitoring, psychoeducation, and behavioral activation. A German trial using health care assistants found benefits comparable to those seen with CC trials.^20^
5. *Psychiatric consultation* should be an accessible and dedicated component of integrated behavioral health care. A common model is for the care manager to meet weekly with a psychiatrist to discuss new patients or those who are not adequately responding to treatment in order to develop patient-specific treatment plans. The psychiatrist will occasionally have one or several in-person or tele-video sessions with selected patients. In other cases, the psychiatrist may recommend and facilitate referral of patients who need more specialized or prolonged psychotherapy or treatment for more complex conditions such as suicidal ideation, substance use disorders, and bipolar disorder or other psychotic disorders. Telepsychiatry can increase access to patients in rural areas or other primary care settings with a shortage of mental health professionals.^21^
6. Although psychotropic prescribing is the most common treatment modality in primary care, *brief psychological therapies* such as motivational interviewing, behavioral activation, or problem-solving treatment are an important component of effectively integrated behavioral health care. Such therapies can be administered in the primary clinic or telephonically by trained behavioral health specialists (e.g., nurse, clinical social worker, psychologist, or other licensed counselor). The therapist can be the care manager or a separate clinician. The number of sessions is typically limited (e.g., eight or fewer), with the most commonly studied treatments in a meta-analysis of 34 trials being cognitive-behavioral therapy (*n* = 13), problem-solving therapy (*n* = 12), and counseling (*n* = 8).^22^ CBT for anxiety had the strongest benefits (pooled effect size of 1.06), whereas the benefits of all three treatments for depression or mixed anxiety and depression were modest (effect sizes of 0.21 to 0.33).

## MODELS OF INTEGRATION

As noted in a 2010 report: “*Collaborative care* and *integrated care* are the two terms most often used to describe the interface of primary care and behavioral health care…Collaborative care involves behavioral health working *with* primary care; integrated care involves behavioral health working *within* and as a part of primary care. In collaborative care, patients perceive that they are getting a separate service from a specialist, albeit one who works closely with their physician. In integrated models, behavioral health care is part of the primary care and patients perceive it as a routine part of their health care.”^8^

The most frequently tested and evidence-based model is *collaborative care* (CC), an approach developed at the University of Washington in which care managers and psychiatric consultants work with primary care physicians to support medication management, provide brief counseling, and coordinate care across clinicians. An analysis of 94 trials of CC for mental health disorders (the most common target being depression) has conclusively demonstrated the superiority of CC compared to usual primary care in improving mental health outcomes.^23^ With its most extensive testing in primary care, CC has also proven effective in selected groups such as pregnant women, adolescents, older adults, neurology and oncology patients, and individuals with chronic pain, diabetes, and other medical disorders.^11^^,^ 24

An *embedded behavioral specialist* model (also known as *co-location)* has been adopted in certain primary practices—especially VA clinics but also in some non-VA primary care settings. This is most often a psychologist who offers consultative services to PCPs and provides on-site care for selected patients, particularly those who may benefit from brief psychotherapy. While popular in some health care systems, there is limited clinical trial evidence that this approach can achieve population-level improvements without paying attention to the core principles outlined in Table 1.^25^ Similarly, *consultation-liaison psychiatry* trials in primary care where a psychiatrist sees patients once or twice for assessment and management advice but does not provide active treatment have not proven this model superior to usual care.^26^

## TYPE OF MENTAL DISORDER

Depression has far and away the most clinical trial evidence: a 2012 Cochrane review of CC for depression and anxiety revealed that most trials (74 of 79) focused on depression.^10^ CC consistently proved superior to usual care with an average effect size of 0.34, which was sustained up to 2 years in the subset of trials examining long-term outcomes. This is in the range of a small (0.2) to moderate (0.5) clinical effect.^27^

Anxiety and related disorders were represented by only five trials in the Cochrane review: two for panic disorder (PD); one for panic disorder and generalized anxiety disorder (GAD); one for posttraumatic stress disorder (PTSD); one for PD, GAD, PTSD, and social anxiety disorder. Effect sizes (0.30) were similar to those seen for depression. A recent primary care trial for PD and GAD showed a similar effect size for of 0.30 for CC.^28^ Additional trials of CC for PTSD have been recently published,^29^ and of six trials, two demonstrated superiority of CC.^30^^,^ 31 These two positive trials had more consistently administered telephone-based psychotherapy, a treatment especially important for PTSD.

Chronic pain is frequently accompanied by comorbid depression or anxiety and shares some features such as stigma, being demanding in terms of time, and benefitting from CBT, self-management, and other nonpharmacological approaches. CC interventions benefit both pain and psychological outcomes in primary care patients with chronic pain.^32^^–^ 35 Although evidence of CC for treating substance use disorders (SUDs) is lacking, studies adapting medication-assisted treatment to primary care settings are ongoing. Also, referral for SUD care can be facilitated in integrated systems, which in turn may improve both SUD and depression outcomes.^36^

Serious mental illnesses (SMIs) such as bipolar disorder and schizophrenia typically require optimal management in the MH specialty setting, although between 10% to 38% of patients with bipolar disorder are treated exclusively in primary care settings.^37^ Patients with SMI who are frequently cared for in public MH settings die as much as 25 years earlier than individuals in the general population, largely as the result of medical causes such as cardiovascular diseases and cancer rather than suicide or accidental death.^38^ Thus, CC intervention where a PCP optimizes the medical care of SMI patients either in the mental health clinic or with closely coordinated visits to primary care is another model of integrated care that may be potentially beneficial.^39^ Although successful interventions have predominantly targeted weight loss and smoking cessation and have been delivered in the psychiatric setting,^40^ one CC trial of medical management in SMI patients had promising results.^38^

A key issue is whether CC can benefit both psychological and medical disease outcomes. A meta-analysis of 24 trials testing integrated care for depression in primary care patients with chronic medical conditions found improved depression outcomes in patients with arthritis, cancer, diabetes, heart disease, and HIV; however, few trials explicitly targeted or assessed outcomes of the medical condition.^24^ Several trials focused on jointly managing depression combined with another condition such as chronic pain,^33^^,^ 41 PTSD,^31^ or diabetes and coronary heart disease^42^^,^ 43 have demonstrated improved outcomes for both depression and the co-managed conditions. Thus, clinical practices should determine whether a care manager is to cover multiple conditions and, if so, whether the scope of practice comprises more than one type of mental disorder or several common mental and medical disorders.^39^

## ADDED BENEFITS FOR MINORITY, UNDERSERVED, AND OTHER VULNERABLE POPULATIONS

Integrated care improves mental health outcomes in all primary care patients but may be especially beneficial in vulnerable populations. The Partners in Care study found greater improvement from CC-type programs relative to usual care for depressed African-Americans and Latinos than for whites in primary care, while the IMPACT study found comparable benefits for minority and white depressed elderly.^44^ The WE Care trial found that a primary care-based intervention in low-income minority women was superior to community mental health referral.^45^

The contribution of contextual (e.g., housing, employment, food insecurity, violence, interpersonal stressors) and culturally relevant factors to mental disorders in vulnerable populations is substantial. A recent trial enhancing collaborations between community agencies and the health care system improved depression outcomes in predominantly minority (46% African-American, 41% Latino) safety-net clients.^44^ The BRIDGE trial showed that standard CC and patient-centered culturally adapted CC had similar benefits on depression outcomes in African-American patients, although patient satisfaction was greater in those receiving the culturally adapted approach.^46^ Another trial found that six telepsychiatry sessions provided by a bilingual psychiatrist to low-income Hispanic primary care patients was superior to usual care in improving depression outcomes.^47^ A potential resource in underserved areas is trained community health workers who can augment primary care services by screening for mental disorders, identifying contextual and cultural factors salient to depression, linking to relevant community resources, and monitoring outcomes.^48^

## COST-EFFECTIVENESS

Of 18 RCT-based economic evaluations of CC, all demonstrated cost-effectiveness estimates below generally accepted thresholds in the US ($15,000–$80 000 per quality-adjusted life-year gained vs. usual care).^23^ Another review of cost analyses performed in 30 CC studies (both RCTs and other designs) concluded that there were more “positive” than “negative” results with regard to reduced health care use, averted productivity loss, and cost-effectiveness.^49^ Although CC benefits in terms of depression outcomes occur in the first year and appear sustained through at least 2 years, there may be slightly higher costs early on.^50^ Actual cost savings may lag behind but can become manifest after 3–4 years. In the IMPACT study, the largest trial of collaborative care to date, health care systems realized over 6 dollars in savings for every 1 dollar spent on CC for depression over a 4-year period^51^ CC must be considered a bundled intervention since meta-regression of trials has not shown any component to be singularly effective or superfluous.^14^ Because illness severity is one factor that predicts CC outcomes,^14^ future research should determine whether stepped care application of CC to more severely ill individuals may be one way of conserving resources; another way is gathering data through automated monitoring.^34^^,^ 41.

## IMPLEMENTATION

Except in a few integrated health care systems, implementation of CC lags far behind the substantial body of clinical trial evidence supporting its use. Model programs include the TIDES program in the VA, the DIAMOND project in Minnesota, and initiatives in Washington, Intermountain Health, and the Department of Defense.^31^^,^ 52 ^–^ 55 One major barrier to wider implementation has been payment systems that have often been segregated into separate reimbursement for medical and mental health (MH) care. The majority of MH-dedicated resources go to only 10% of all patients with MH disorders; 90% of patients with MH disorders are seen in the medical sector, and two-thirds receive no treatment for their MH disorder.^56^ Moreover, 20% to 40% of the total health budget is used to provide medical services for patients with MH disorders. Untreated MH disorders are associated with higher medical illness complication and treatment nonresponse rates, increased health care use and costs, and premature death. In 2012, the annual additional cost of medical care for the nearly 41 million Americans with MH conditions was an estimated $290 billion.^13^ Therefore, integrated care for MH disorders may jointly benefit medical and mental health outcomes, an important consideration when aligning services and payments in accountable care organizations (ACOs).

Another barrier to implementation is a workforce inadequately trained in providing integrated, team-based care.^57^ The American Psychiatric Association has a grant from CMS to train 3500 psychiatrists in CC. Surveys have indicated CC teamwork can be rewarding for both psychiatrists^58^ and primary care physicians^59^ and have highlighted psychiatrists’ educational needs.^60^ The American Medical Association has recently approved CPT codes for collaborative care that will be available in 2017 or 2018. ACO and Patient Centered Medical Home (PCMH) initiatives might also facilitate dissemination of CC,^61^ particularly since the National Committee for Quality Assurance has proposed measures of depression screening and remission as key quality indicators. However, two limitations of initial ACO guidelines should be addressed for optimal CC delivery.^62^ First, psychiatrists are the only MH professional defined by CMS as participating ACO clinicians, to the exclusion of social workers, psychologists, and other health professionals who may serve as care managers and behavioral health consultants in integrated settings. Second, only one of the 65 quality measures proposed for ACOs pertain to mental health care (depression screening) while no performance incentives or standard billing codes are tied to fundamental CC components such as decision support, measurement-based care, or registry maintenance.

Ways in which CC is implemented can vary. In some cases, care managers and psychiatric consultants work for the primary care clinic or health care system. In others, they may be contracted from a community mental health center or other specialty mental health programs. Care managers may be full time (in larger practices) or work part-time and provide services to several smaller practices. Psychiatric consultants often cover and consult to more than one primary care practice. Payment for these programs also varies, from salaried payment in fully capitated systems to case rate payments in managed Medicaid programs to fee-for-service payments using newly established CMS billing codes.

Table 2 lists some practical suggestions for smaller practices lacking the resources of larger integrated health systems. Because RCTs have typically tested CC as a bundled intervention, the impact of the individual strategies outlined in Table 2 is less certain. Nonetheless, practices in which all the components of CC are not yet feasible might find some of these strategies useful.
